# Respiratory symptoms and lung function 8–10 months after community exposure to chlorine gas: a public health intervention and cross-sectional analysis

**DOI:** 10.1186/1471-2458-13-945

**Published:** 2013-10-09

**Authors:** Kathleen A Clark, Debjani Chanda, Pallavi Balte, Wilfried J Karmaus, Bo Cai, John Vena, Andrew B Lawson, Lawrence C Mohr, James J Gibson, Erik R Svendsen

**Affiliations:** 1 University of South Carolina, Columbia, SC, USA; 2 University of Memphis, Memphis, TN, USA; 3 University of Georgia, Athens, GA, USA; 4 Medical University of South Carolina, Charleston, SC, USA; 5 South Carolina Department of Health and Environmental Control, Columbia, SC, USA; 6 Tulane University, New Orleans, LA, USA

**Keywords:** Chlorine gas, Irritant gas, Respiratory symptoms, Graniteville, Environmental disaster, Sensitivity, Specificity, Respiratory questionnaire, Spirometry

## Abstract

**Background:**

We implemented a community based interventional health screening for individuals located within one mile of a 54 metric tons release of liquid chlorine following a 16 tanker car train derailment on 6 January, 2005 in Graniteville, South Carolina, USA. Public health intervention occurred 8–10 months after the event, and provided pulmonary function and mental health assessment by primary care providers. Its purpose was to evaluate those exposed to chlorine for evidence of ongoing impairment for medical referral and treatment. We report comparative analysis between self-report of respiratory symptoms via questionnaire and quantitative spirometry results.

**Methods:**

Health assessments were obtained through respiratory symptom and exposure questionnaires, simple spirometry, and physical exam. Simple spirometry was used as the standard to identify continued breathing problems. Sensitivity, specificity, positive and negative predictive values were applied to evaluate the validity of the respiratory questionnaire. We also identified the direction of discrepancy between self-reported respiratory symptoms and spirometry measures. Generalized estimation equations determined prevalence ratios for abnormal spirometry based on the presence of participant persistent respiratory symptoms. Covariate adjustment was made for participant age, sex, race, smoking and educational status.

**Results:**

Two hundred fifty-nine people participated in the Graniteville health screening; 53 children (mean age = 11 years, range: <1-16), and 206 adults (mean age = 50 years, range: 18–89). Of these, 220 (85%) performed spirometry maneuvers of acceptable quality. Almost 67% (n = 147) displayed abnormal spirometry, while 50% (n = 110) reported persistent new-onset respiratory symptoms. Moreover, abnormal spirometry was seen in 65 participants (29%) who did not report any discernible breathing problems. This represented a net 16.8% underreporting of symptoms. Sensitivity and specificity of questionnaire self-report of symptoms were low at 55.8% and 61.6%, respectively. Persistent cough (41%) and shortness of breath (39%) were the most frequently reported respiratory symptoms.

**Conclusion:**

Eight to ten months after acute chlorine exposure, the Graniteville health screening participants under-reported respiratory symptoms when compared to abnormal spirometry results. Sensitivity and specificity were low, and we determined that relying upon the self-report questionnaire was not adequate to objectively assess the lung health of our population following irritant gas exposure.

## Background

Public health strategies customarily concentrate on the effective use of recovery efforts and the mitigation of persistent health problems through community monitoring, education, and intervention after community based disasters [1]. Nonetheless, survivors of large man-made disasters such as the 1945 Hiroshima-Nagasaki nuclear attacks, the 1984 Bhopal Union Carbide methylcyanite gas release, the 1986 Chernobyl Nuclear core meltdown, and the many irritant gases and fine particles released during the 2001 World Trade Center attack, have all reported continuing health consequences years after these events [2-8]. This is of concern, because long-term lung problems such as reactive airway dysfunction syndrome (RADS) or irritant induced asthma (IIA) have been shown to develop even after a single irritant or toxic gas exposure [9-15].

Chlorine is one of the most commonly manufactured industrial chemicals which at standard temperature and pressure exists as an irritant gas [16]. It has a broad range of uses from waste water treatment to household cleaning products [16]. When depressurized and released, chlorine gas lingers at ground level and is not readily dispersed into the atmosphere [16-18]. Chlorine accidents are quite common and thousands of people are collectively exposed each year [17-26]. Approximately 18% of accidental releases occur during transport, and though large events are relatively rare, they are not unusual [23,27,28].

On 6 January, 2005, a 16 car train derailment led to a single tanker car breach and an estimated 54,422 kg release of chlorine in Graniteville, South Carolina [29]. Over 5,000 residents were evacuated within one mile of the accident [29]. Nine deaths were initially reported, 71 individuals hospitalized, and at least 529 people were treated and released from local emergency departments [30,31]. In total, there were 1,384 known casualties [31].

Within 6 months of the accident, the South Carolina Department of Health and Environmental Control (SCDHEC) developed and sustained a voluntary health registry hotline to identify community members continuing to suffer adverse effects following chlorine exposure [31]. Although many of those most severely exposed were already receiving adequate primary and follow-up care; there were others in the community that were concerned about their health status and requested that SCDHEC perform a public health screening. The main objective was to provide public health service to those who did not have adequate health care coverage but were exposed to chlorine gas. With this effort, we were able to apply community based participatory research practices while implementing scientific research through the Graniteville Recovery and Chlorine Epidemiology project (GRACE). Specific details regarding this process have been reported previously [31].

Self-report of symptoms questionnaires are common public health tools used to assess continuing health effects suffered by disaster populations. Questionnaires serve to identifying those potentially suffering from ongoing health problems. However, we need to consider the existence of differential misclassification when individual exposure measurements are not available [32]. Current paradigms suggest that individuals suffering from such tragic events will actually over-estimate their degree of health impairment [32]. Multiple factors including posttraumatic stress disorder (PTSD) may contribute to this recall bias and potentially skew results [32-35].

The purpose of this paper was to report results from a community health screening that occurred 8–10 months after the 2005 Graniteville train accident. As Graniteville psychological impacts have been previously described in detail elsewhere [33]; we focused on the validity of our respiratory questionnaire in comparison to spirometry assessment. We wanted to know if our respiratory symptoms questionnaire adequately captured and identified individuals who continued to have persistent respiratory difficulties following chlorine exposure.

## Methods

### Health screening participants

Anyone exposed to chlorine was instructed to call a state sponsored hotline for inclusion into the SCDHEC health registry established as a result of the disaster. Upon telephone interview, we mapped and determined caller locations at the time of chlorine release. Anyone who lived in, worked in, responded to, or traveled within one mile of the train derailment at the time of the accident was then invited to participate in the GRACE health screening.

### GRACE health screening questionnaire and assessment

Between 8–10 months after the disaster, GRACE health screening participants were interviewed regarding any persistent physical or psychological impairment since the time of the accident. Pulmonary disease was queried using the standardized and previously validated American Thoracic Society 1978 Adult Lung Disease (ATS-DLD-78) questionnaire [36]. This established questionnaire has been recommended for use in epidemiologic studies where the prevalence of chronic respiratory symptoms and disease are being assessed [36]. We chose to implement the investigator-led design, taking special attention to prevent any suggestive influence on participants. We also obtained information regarding newly diagnosed conditions, exacerbation of pre-existing disease, family history of asthma or other pulmonary disease, and pre-existing respiratory allergies. Specific respiratory symptoms such as new-onset and persistent cough, shortness of breath, wheezing, or chest tightness were considered. Questions were formatted for “yes” or “no” responses such as: “Did you have any respiratory symptoms that started after the train accident? Did you have: Coughing? Wheezing or whistling in your chest? Shortness of breath? Chest tightness?”.

### Physical assessment

Physical examination by licensed primary care practitioners was performed for each participant. This included a medical history and general exam for signs and symptoms of physical impairment. Current medications, as well as those used before and immediately following the accident were reviewed.

### Spirometry screening

Spirometry screenings were performed using current American Thoracic Society/European Respiratory Society Guidelines (ATS/ERS) [37]. All spirometry technicians were National Institute of Occupational Safety and Health (NIOSH) certified and state licensed respiratory therapists. Parameters of interest were forced vital capacity (FVC), forced expiratory volume at one second (FEV1), the FEV1/FVC ratio, and the FEF mid quartile average-flow (FEF _25–75_). Each participant performed at least three acceptable maximal forced expiratory maneuvers. The largest FEV1 and FVC were used for the FEV1/FVC ratio [37]. We assessed air-flow limitation reversibility using standard dose administration of a short acting β-agonist inhaled bronchodilator. Additional post-bronchodilator spirometry was then performed as outline by the 2005 ATS/ERS Task Force [37]. Participants with abnormal spirometry or who were unable to perform acceptable spirometry maneuvers because of persistent pulmonary symptoms were referred for pulmonary consult.

The 3^rd^ National Health and Nutritional Examination Survey (NHANESIII) regression coefficients were used to estimate percent predicted and lower limit of normal (LLN) spirometry values [38-41]. An abnormal LLN is indicative of the lowest 5^th^ percentile of normal subjects being classified as “abnormal” [39]. We chose LLN, instead of percent predicted as our reference classification because it is a more conservative method and is known to produce the least number of false positive spirometry results [39,40]. We utilized the Global Initiative for Chronic Obstructive Lung Disease classification to identify severity of obstructive limitation [42]. Flow-volume and volume-time tracings were examined to determine maneuver quality and repeatability for each individual. Only tracings that met ATS/ERS 2005 spirometry guidelines were analyzed [37].

### Data analysis

Frequency tables were constructed for GRACE health screening demographics, symptoms, and spirometry results. Demographic differences between GRACE health screening participants, those enrolled in the SCDHEC health registry, and chlorine exposure victims that received medical intervention within one week of the accident were determined using Chi-square with multiple comparison correction.

We defined abnormal spirometry as having FEV1, FVC, or FEV1/FVC ratio below predicted LLN. A FEV1/FVC ratio below LLN was considered to represent an obstructive spirometry pattern. Severity of obstruction was identified for those with obstructive limitation. Severity levels were: Mild: FEV1 % predicted > 80%, Moderate: 50% < FEV1 < 80%, Severe: 30%< FEV1 <50%, and Very Severe FEV1: <30% predicted [39,42]. Having FEV1 *and* FVC parameters below LLN with a FEV1/FVC ratio > LLN, was considered a restrictive air-flow pattern [39,40]. LLN estimates were derived from ATS recommended NHANES III predicted values and adjusted for age, gender, race, and height [37,39]. Air-flow reversibility was not considered, as our primary objective was to compare persistent new-onset respiratory symptoms to spirometry results 8–10 months after the chlorine release.

Persistent new-onset respiratory symptoms were defined as any cough, wheezing, shortness of breath, or chest tightness first experienced after the chlorine spill and persisting up until the GRACE health screening. Generalized estimation equation procedures with the log-binomial were used to estimate adjusted prevalence ratios for abnormal spirometry parameters based upon respiratory symptoms. Covariate adjustment was made for participant age, sex, race, smoking and educational status.

A two by two contingency table was constructed for participants comparing respiratory symptoms with spirometry results. From this, we were able to determine the proportion of under or over-reporting of symptoms by comparing differences between discordant pairs. Positive and negative predictive values, sensitivity, and specificity measures were also determined. All analyses were performed using the SAS 9.2 software program (SAS Institute; Cary, NC USA).

### Ethical considerations

University of South Carolina and SCDHEC Institutional Review Board approvals were obtained prior to the community public health intervention. Both review boards ruled that the intervention was not human subjects research. However, participant informed consent or assent was obtained prior to each individuals’ health screening. Additionally, SCDHEC facilitated follow up care or referral for detected clinical conditions to local primary care providers.

## Results

**Figure 1:**
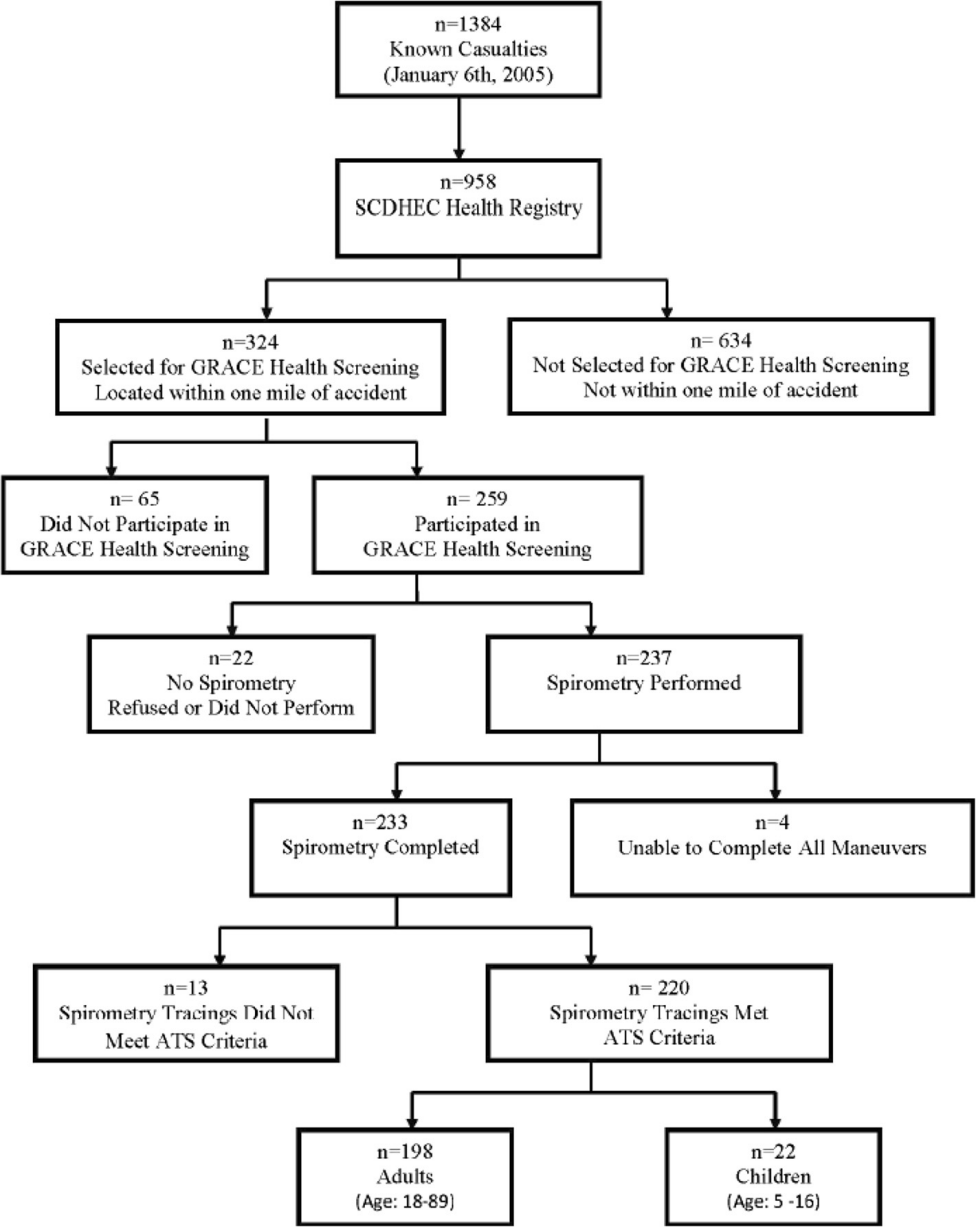
Known casualties from the Graniteville train derailment and progression to participants of the GRACE health screening.

Of the 958 enrolled in the SCDHEC health registry, 324 individuals were located within one mile of the chlorine accident and asked to participate in the GRACE health screening. Nearly 80% of those eligible (n = 259) participated (Figure 1). Forty-four GRACE health screening participants were children whose age was between 5–16 years (mean age = 11 years), with an additional nine participants under five years of age. There were 206 adults who participated between the ages of 18–89 years (mean = 50 years) (Table 1). The mean age for GRACE health screening participants was 47.4 years, and screened participants tended to be older than those who received medical care within one week of the accident (mean = 38.2 years), or for all individuals enrolled in the SCDHEC health registry (mean = 41.6 years) (Table 1).

**Table 1:** Demographic profile of the 2000 Graniteville Census population versus GRACE health screening participants versus those that participated in the SCDHEC voluntary health registry

**Demographic characteristics**	**Immediate medical care N (%)**	**GRACE health screening vs. immediate medical care p-value**	**GRACE health screening N (%)**	**GRACE health screening vs. SCDHEC registry p-value**	**SCDHEC registry N (%)**
N	860		259		958
Mean age (years)	38.2 (19.5)		47.4 (20.0)		41.6 (22.6)
Under 5	43 (5.1)	0.0114	9 (3.5)	0.0534	42 (4.4)
5-17	88 (10.4)	<0.0001**	44 (17)	0.0250	196 (20.5)
18-65	648 (76.9)	<0.0001**	153 (59)	0.0152	569 (59.4)
Over 65	64 (7.6)	<0.0001**	53 (20.5)	0.0308	150 (15.7)
Gender					
Female	371 (45.3)	<0.0001**	114 (55.6)	0.0159	489 (51)
Male	448 (54.7)	<0.0001**	115 (44.4)	0.0165	469 (49)
Race					
White	433 (61.2)	0.5801	165 (63.6)	0.0139	661 (68.9)
Black	237 (33.5)	0.0470	82 (31.8)	0.0240	245 (25.6)
Hispanic/other	36 (5.1)	0.0699	12 (4.6)	0.0481	53 (5.5)
Education					
< 9^th^ Grade	109 (15.4)	<0.0001**	27 (10.3)	0.0376	95 (9.9)
Some high school	138 (19.5)	0.6999	52 (20.1)	0.0258	196 (20.5)
High school grad	276 (39.0)	0.7191	103 (39.7)	0.0182	394 (41.1)
College/2 yr	129 (18.2)	0.0135	57 (22.1)	0.0281	177 (18.5)
Bachelors	47 (6.6)	0.1421	14 (5.4)	0.0420	67 (7)
Post-graduate	8 (1.1)	0.0198	6 (2.5)	0.0654	28 (2.9)

**Significant with Bonferroni multiple comparison correction = 0.0033 (α = 0.05).

P values represent Chi-square results between demographic groups.

All groups were similar in racial composition; each consisted of at least 60% Caucasian, 25% African American, with approximately 5% from other racial groups (Table 1). Nonetheless, significant demographic differences were seen between GRACE health screening participants and those who received prompt medical care. Those that received care were on average younger, between 18–65 years of age, and more likely to be male (Table 1). Proportionally, GRACE health participants consisted of more females, children, and senior citizens (Table 1). Over 75% of those who received prompt medical care were between 18 to 65 years of age, compared to only 59% for the GRACE health screening group (Table 1). No discernable demographic differences were observed between GRACE health screening participants and those enrolled in the SCDHEC voluntary health registry (Table 1).

### All symptoms report

Trouble breathing, eye problems, and problems of the ears, nose, and throat (ENT) were the three most common general complaints (Table 2). Dermatological, gastrointestinal, and vertigo symptoms were also reported by over 10% of participants (Table 2). Worsening of pre-existing conditions was reported by 15 individuals (5.8%). Eighteen percent (n = 47) of the 259 GRACE health screening participants were previously diagnosed with chronic pulmonary disease prior to the train accident, and seven children and 24 adults were known asthmatics (Table 2). The remainder (n = 15), were previously diagnosed with chronic obstructive pulmonary disease (COPD). One adult had been previously diagnosed with byssinosis from working in the local textile industry.

**Table 2:** Most common symptoms reported by all participants (n = 259) at the time of the GRACE health screening

**Symptoms**	**Total (n = 259)**		**Adults (n = 206)**		**Children (n = 53)**	
	**N**	**%**	**N**	**%**	**N**	**%**
Breathing problems	119	45.9	110	50.9	9	20.9
ENT problems	84	32.4	72	33.3	12	27.9
Eye problems	76	29.3	64	29.6	12	27.9
Skin problems	36	13.9	27	12.5	9	20.9
Nausea/vomiting/diarrhea	35	13.5	30	14.0	5	11.6
Vertigo	32	12.4	26	12.0	6	13.9
Previously diagnosed pulmonary disease	47	18.1	40	19.4	7	13.2
Asthma	31		24		7	
COPD	15		15		-	
Byssinosis	1		1		-	
Worsening of pre-existing conditions	15	5.8	14		1	

### Newly diagnosed respiratory conditions and symptoms

The most frequently reported new-onset respiratory symptom was cough (Table 3). Over 72% of participants had adverse respiratory symptoms at the time of the chlorine spill, whereas, 50% complained of persistent respiratory symptoms at the time of the GRACE health screening. Persistent new-onset wheezing, shortness of breath, and chest tightness were reported by between 30 -39% of participants (Table 3).

**Table 3:** Frequency (%) of new-onset respiratory symptoms reported during the GRACE health screening and at the time of chlorine exposure (N = 220)

	Persistent new-onset respiratory* symptoms at time of GRACE health screening	**Respiratory symptoms at time of chlorine spill**
	**N = 110 (50%)**	**N = 158 (72.5%)**
Cough	91 (41.4)	126 (57.3)
Wheeze	71 (32.3)	88 (40.0)
Shortness of breath	86 (39.1)	111 (50.5)
Chest tightness	68 (30.1)	95 (43.2)

*Reported 8–10 months after chlorine exposure, and represented only new-onset respiratory complaints consistently experienced since the chlorine spill.

Eighty three participants (32%) received a newly reported chronic respiratory diagnosis after the chlorine spill (Table 4). Bronchitis, and asthma were the most common conditions diagnosed among the participants (Table 4). New onset asthma was diagnosed in 18% (n = 13) of participants. All participants with newly diagnosed chronic conditions were adults, and except for those diagnosed with emphysema, were more likely to be nonsmokers (n = 10) (Table 4).

**Table 4:** New-onset physician diagnosis respiratory conditions following the 2005 chlorine spill by GRACE health screening participants and stratified by participant smoking status (N = 259)

**Newly diagnosed respiratory conditions**	**N (%) 83 (32%)**	**Smoker**	**Nonsmoker**
Acute bronchitis	23	6	17
Hay fever/nasal allergies	17	1	16
Asthma	13	3	10
Rhinitis/sinusitis	13	3	10
Pneumonia	10	1	9
Chronic bronchitis	4	1	3
Emphysema	3	2	1

### Spirometry

Simple spirometry was performed for 237 (92%) screened participants (Figure 1). Four adult participants attempted spirometry, but were unable to complete the forced breath maneuvers. Spirometry tracings from 13 participants (8 adults; 5 children) did not meet the 2005 ATS/ERS criteria for quality and repeatability and were removed prior to analysis. In total, 220 spirometry screenings were acceptable; 198 adults and 22 children.

One hundred twenty-six individuals (68%) had a markedly reduced FEV1, as shown by an abnormal LLN value (Table 5). There were 20 (9.19%) participants who revealed *both* an abnormal FEV1 with a significantly reduced FEV1/FVC ratio (Table 5). Fifteen of these met criteria for moderate to severe obstructive air-flows (nine complained of persistent respiratory symptoms), whereas, two participants displayed very severe obstructive air-flows and were without any persistent respiratory symptoms [39,42]. In total, 80 (36%) participants had both FEV1 and FVC below LLN, with their FEV1/FVC ratio > LLN; suggestive of a possible restrictive air-flow pattern (Table 5) [39].

**Table 5:** Spirometry results with PFT pattern classification based upon new-onset respiratory symptoms (N = 220)

	**Mean NHANES % predicted (std)**	**Below LLN and symptoms N = 110 (%)**	**Below LLN and no symptoms N = 110 (%)**	Adjusted prevalence ratio*** (95% CI)	***p-value***
	**Median**				
FEV1	72.1 (21.5)	75 (68.2)	51 (46.4)	1.73 (1.11, 2.69)	0.015*
	74.0				
FVC	81.7 (23.6)	61(55.5)	49 (44.6)	1.66 (1.0, 2.77)	0.051
	80.0				
FEV1/FVC ratio %	82.4 (0.09)	15 (13.6)	9 (8.2)	2.25 (0.542, 9.34)	0.264
	84.3				
FEF_25-75%_	95.1 (39.1)	16 (14.6)	9 (8.2)	1.60 (0.50, 5.16)	0.431
	93.2				
Any abnormal parameter		82 (74.5)	64 (59.1)	1.62 (1.10, 2.39)	0.015*
Air-Flow pattern results**	Normal	28	45		
	Obstructive^§^	12	8		
	Mild	3	0		
	Moderate	4	3		
	Severe	5	3		
	Very severe	0	2		
	Restrictive^§§^	46	34		
	FEV1<LLN^§§§^	24	23		

*Statistically significant at α = 0.05.

**Normal = FEV1 and FVC ≥ LLN and FEV1/FVC ratio ≥ LLN.

§Obstructive Spirometry Pattern = FEV1/FVC ratio < LLN.

Mild: FEV1% predicted > 80% ,Moderate: 50% < FEV1 < 80% predicted, Severe: 30%< FEV1 <50% predicted, Very Severe: <30% predicted [39,42].

§§Restrictive Spirometry Pattern = FEV1, FVC, and FEV1/FVC Ratio > LLN.

***Covariate adjustment for age, sex, race, smoking and educational status.

Adjusted prevalence ratios (PR), 95% confidence intervals, and Chi-square p-values for the association between report of respiratory symptoms and spirometry parameters below LLN.

### Respiratory questionnaire accuracy

Those participants that complained of ongoing respiratory symptoms were significantly more likely to display an FEV1 below LLN (PR = 1.73, 95% CI: 1.11, 2.69). Also, health screening participants with persistent respiratory symptoms were 60% more likely to have at least one abnormal spirometry parameter than those without persistent symptoms (PR = 1.62 95% CI: 1.10, 2.39) (Table 5). However, sensitivity and specificity of the respiratory symptoms questionnaire were low at 55.8% and 61.6%, respectively (Table 6). Report of symptoms was a moderate predictor for abnormal spirometry; displaying a 74.5% positive predictive value (Table 6). But those participants without symptoms often had abnormal spirometry (NPV 59.1%). Observed differences between reported respiratory symptoms and abnormal spirometry results, revealed a net 16.8% underreporting of symptoms when discordant pairs were examined (Table 6).

**Table 6:** Comparison between persistent new-onset respiratory symptoms and abnormal spirometry based upon participant lower limit of normal estimates (LLN)

	Abnormal* spirometry	**Normal spirometry**	**Predictive values**
	**N = 145 (%)**	**N = 94 (%)**	
Persistent new-onset respiratory symptoms**	82 (55.9)	28 (38.7)	**PPV**
			74.5%
**No pulmonary symptoms**	65 (44.1)	45 (61.3)	**NPV**
			59.1%
**Validity measures**	**Sensitivity**	**Specificity**	
	55.8%	61.6%	

*Abnormal spirometry FEV1, FVC, or FEV1/FVC Ratio below NHANES % predicted LLN.

**Reported symptoms were persistent new-onset cough, wheeze, shortness of breath, or chest tightness since January 2005, Graniteville chlorine spill.

## Discussion

The self-report symptoms questionnaire did not adequately represent the current lung function status of GRACE health screening participants. Sensitivity and specificity measures were low, indicating marginal accuracy. Abnormal spirometry was seen in 65 participants (29%) who did not report any discernible breathing problems. This represented a net 16.8% underreporting of symptoms. Due to the traumatic and potential litigious nature of the event, we expected an increased report of respiratory symptoms from participants in comparison to spirometry findings [35]. We also surveyed exposed individuals 8–10 months after exposure and asked them to identify their first occurrence of respiratory symptoms. Recall bias suggests that those with known disease or exposure are more likely to recall symptoms rather than to forget the existence of symptoms [32,43]. Our health screening participants did show evidence for panic and post traumatic stress disorder [33]. Yet, persistent respiratory symptoms were underreported compared to spirometry; even with 32% (n = 83) of those screened receiving a newly diagnosed chronic respiratory condition such as asthma, chronic bronchitis, or sinusitis. Solely relying upon the self-reported ATS-DLD respiratory questionnaire during post-disaster public health screening would have underestimated the degree of post-disaster pulmonary impairment.

Over-reporting of symptoms following disasters may be more specific to the type of disaster and the characteristics of the affected disaster population. Although purely speculative, symptom over-reporting would suggest that those affected might benefit from continued symptoms. Such benefits could be in the form of improved access to health care, financial compensation, or improved psychosocial state. However, an under-reporting of symptoms is equally as plausible. When a disaster population is disenfranchised, with no likelihood for remediation, then it may be a simple coping mechanism to just “ignore the obvious” and minimize the disaster and its impact.

The population of Graniteville was established as a result of the regional textile industry. They are hardworking individuals who relied upon the local mill for their livelihood and health benefits. Because the accident occurred at the mill, it also suffered, and fought to remain in production in the immediate months after the disaster. Employees were expected to report for work when the mill reopened 14 days after the accident. As the welfare of the entire community benefited if the mill remained open, it may have benefited workers and their families to say that they were “healthy” and thus minimize the reported health impacts from the disaster. Nevertheless, reliance on self-report of symptoms did not adequately capture the respiratory status of those who participated in the GRACE health screening. There may be other, yet determined, explanations for our results. Loren (1993), suggested that the best way to screen individuals for respiratory impairment was to use a combination of questionnaire, physiological investigation, and clinical judgment [44]. Our findings reinforce this suggestion.

As with most disasters, we did not have pre-exposure health records for our screened participants and cannot unequivocally identify chlorine exposure to be the causal factor for the high prevalence of lung function impairment. However, previous diagnosis of chronic pulmonary disease (18.2%), participant report for worsening of previously diagnosed pulmonary conditions (5.8%), and newly diagnosed chronic respiratory disease (32%) were each independent strong predictors for abnormal lung function (data not shown).

Almost 67% (n = 147) of those screened displayed abnormal spirometry, whereas, 50% (n = 110) reported persistent new-onset respiratory symptoms. Recall that one rationale for screening is to help identify asymptomatic individuals who would benefit from further evaluation [43]. Of the 259 participants, only 22 (8.5%) received emergency treatment on the day of the accident. During the immediate disaster decontamination and triage, most of the GRACE health screening participants did not display symptom severity to warrant emergent medical care. Therefore, most were classified as having mild to moderate initial symptoms. However, more than half of those screened exhibited some level of abnormal lung function 8–10 months after the disaster.

The 2007–2010 NHANES survey (n = 9,024) reported that approximately 80% of the general US population displayed normal lung function [40]. An obstructive air-flow pattern was seen in 13.5% of the population, with the majority exhibiting mild obstruction (7.5%), and most others exhibiting moderate obstruction (5.4%) [40]. Only 0.7% exhibited severe obstruction [40]. These findings are in stark contrast to the spirometry results of the GRACE health screening participants. Less than 50% (n = 73) of our participants demonstrated normal air-flow patterns. We observed 20 individuals (9.1%) who had some degree of obstructive air-flow, and 15 of those individuals had been previously diagnosed with emphysema or chronic bronchitis. However, the severity of obstruction far exceeded those of the general population [40]. Seventy-five percent of those with an obstructive pattern (n = 15) displayed moderate to severe obstruction, and two individuals showed evidence of very severe obstruction. Because we did not have access to pre-exposure lung function measures, we could not determine whether chlorine exposure exacerbated the severity of disease.

More remarkable was the prevalence of restrictive air-flow pattern. We understand that spirometry testing cannot conclusively identify restrictive lung disease, but our findings are suggestive that additional follow-up was warranted. In comparison to the general US population (restrictive pattern = 6.5%), our 36.4% (n = 80) occurrence appeared to be well beyond random variation between screening populations. This is an important finding, as restrictive lung disease has been associated with a substantially higher risk of death (HR 1.7 95% CI 1.4 – 2.0) when compared to individuals with normal lung function [45].

The most frequently reduced single parameter was FEV1<LLN (n = 126), followed by a reduction in FVC <LLN (n = 110). Often, these reductions were observed in conjunction with each other. However, FEV1<LLN alone, was seen in 47 participants. An isolated reduction in FEV1 cannot diagnose any distinctive abnormal air-flow pattern. Nevertheless, in previous population based studies, reduction in only FEV1 was a reliable predictor of mortality from chronic respiratory or cardiovascular disease [46-48]. In addition, a recent Asian study found that isolated FEV1 reduction was associated with a history of smoking, abnormal chest radiography, and history of asthma or chronic bronchitis [49].

We found that a well established, standardized respiratory questionnaire was unable to capture the high frequency of air-flow impairment observed within our GRACE health screening population. When respiratory symptoms were reported, the risk of having abnormal FEV1 and FVC were significantly greater (PR adj = 1.73 and PR adj = 1.66, respectively) than those individuals reporting no symptoms (Table 5). However, sensitivity, specificity, and negative predictive values for the respiratory questionnaire had low to fair accuracy for predicting abnormal spirometry (Table 6). The best predictive capability was observed with FEV1.

Others have also investigated lower respiratory symptoms and their association with spirometry results. In a multi-clinic based investigation of 200 patients with known respiratory illness, specificity was high (83-95%), negative predictive value was moderate (71-74%), but sensitivity and positive predictive values were poor (12-40%) for the 1978 ATS-DLD respiratory questionnaire for both restrictive and obstructive air-flow patterns [48]. Jones et al. (1986), reported that “formal analysis of symptoms failed to produce useful information” when they compared symptoms to spirometry for 60 adults, acutely exposed to chlorine [50]. In 252 victims exposed to methyl isocyanate (MIC) during the 1984 Bhopal gas disaster, there was a positive association between the presence of lower respiratory symptoms and mean annual rate of FEV1 decline [51]. Varied results have also been observed for the different cohorts studied after the World Trade Center disaster (WTC). In a retrospective cohort study of residents located within the WTC exposure zone, Reibman et al. (2013), compared new-onset and persistent new-onset symptoms to spirometry [52]. They found no univariate association between symptoms and spirometry results [52]. However, in another WTC study, exposed firefighter FEV1 volume and lower respiratory symptom recovery was significantly associated [53]. Skloot et al. (2004) did not find any association between the prevalence of lower respiratory symptoms and spirometry results, but symptoms and forced oscillometry were more closely correlated in WTC ironworkers [54]. As with the WTC firefighters, our best predictive capability came with the association between respiratory symptoms and FEV1 below LLN for our screened chlorine victims.

### Limitations

We limited our inclusion criteria to people located within one mile of the accident site, but did not have personal chlorine exposure estimates for each participant at the time of the intervention. Established plume models estimated this area to have had extremely high concentrations of chlorine gas (>400 ppm) [55]. Nonetheless, exposure may have been differential over the area; as wind dispersion, topography, and indoor sheltering could have affected the duration or intensity for each participant. Also, some people immediately evacuated Graniteville, whereas, others were known to have “sheltered in place” indoors for up to eight hours before evacuation. Additional analysis using estimates of personal exposure to chlorine would be valuable.

Of the 53 children who participated in the health screening, only 22 spirometry maneuvers met ATS criteria. Participant age and the inability to follow instructions to perform proper technique most likely contributed to the reduction in usable spirometry measures. Regardless, strong conclusions regarding the lung health of children exposed to chlorine could not be ascertained. Further investigation utilizing quantitative measures to specifically assess the children who were exposed to the chlorine could be extremely informative.

It is unknown what other irritant exposures may have contributed to our results. Because screening was performed in the early to late fall, seasonal allergies may have played some role [35]. Furthermore, we do not have information regarding second-hand smoke exposure or adherence to prescribed medications.

### Strengths

One strength of our analysis was our *‘a priori’* decision to obtain reliable and accurate spirometry maneuvers. As quality maneuvers are *both* strongly technician-technique and patient effort dependent, caution must be taken to assure quality [37]. All clinicians performing spirometry were certified by NIOSH and state licensed respiratory therapists. Furthermore, we performed reviews of flow-volume and volume-time spirometry graphs prior to participant inclusion into the analytical dataset. Only spirometry maneuvers that met 2005 ATS/ERS guidelines for reliability and reproducibility were included in our analysis [37]. Hence, we believe our sensitivity and specificity estimates were accurate for our comparison between the respiratory symptoms questionnaire and spirometry results.

Through out this intervention, we employed the basic premise of community based participatory service by placing our emphasis on service and the needs of the community rather than investigative research [31,56]. This approach provided helpful information for health screening participants regarding their pulmonary health. However, we were also able to assess whether the sole use of a self-reported respiratory questionnaire could accurately determine participant lung function status. Additional studies are needed to ascertain the association between chlorine exposure and decreased lung function.

## Conclusion

Self-report of respiratory symptoms via the ATS-DLD questionnaire did not provide an adequate stand-alone respiratory health screening tool in our disaster population. Nonetheless, the presence of persistent respiratory symptoms was moderately predictive for identifying abnormal lung function; but the lack of respiratory symptoms was not predictive of normal spirometry. Specific to the Graniteville population, an underreporting of symptoms was seen 8–10 months after a single chlorine gas exposure. We recommend that repeated, longitudinal, quantitative health monitoring with appropriate professional consult be provided to truly mitigate persistent health problems following such disasters.

## Abbreviations

>: Greater than; <: Less than; 95% CI: 95% Confidence Interval; ATS: American Thoracic Society; ATS/ERS: American Thoracic Society/European Respiratory Society; COPD: Chronic obstructive pulmonary disease; ENT: Ears, nose, and throat; FEV1: Forced expiratory volume at one second (Liters); FEV1/FVC ratio: Proportion of forced expiratory volume at one second and forced vital capacity × 100 (%); FVC: Forced vital capacity; GRACE: Graniteville recovery and chlorine epidemiology; IIA: Irritant induced asthma; LLN: Lower limit of normal; NPV: Negative predictive value; NHANESIII: The 3rd National health and nutritional examination survey; NIOSH: National Institute of Occupational Health and Safety; Ppm: Parts per million; PPV: Positive predictive value; PTSD: Posttraumatic stress disorder; PR: Prevalence ratio; RADS: Reactive airways disorder syndrome; SAS: Statistical analytics software.

## Competing interests

The authors declare that they have no competing interests.

## Authors’ contributions

KC: Primary author of manuscript, statistical analysis, reviewed spirometry graphics and questionnaire quality, clinical interpretation of findings, entered and reviewed data, managed database, data interpretation, and corresponding author. DC: Early manuscript preparation, entered data, analyzed data, and managed data. PB: Data review, data entry, data analysis, data interpretation, Table and Figure preparation. BC: Statistical analysis design, data interpretation, authored part of methods section. WJK: Initial intervention, health screening design, statistical design, data analysis design and interpretation, manuscript formatting, mentoring of writers, consulting on data analysis. AL: Initial concept, health screening and study design, especially in reference to statistical design and data analysis planning. JV: Initial intervention, health screening design, with special focus towards epidemiological design, data interpretation, reporting design, manuscript formatting, mentoring of the team. LM: Clinical interpretation, consulted on clinical data interpretation and reporting of findings. JJG: Initial concept of public health intervention , data collection and screening of community members, study design, clinical data interpretation. All authors reviewed and commented on manuscript prior to submission. ERS: Principle investigator, South Carolina State Environmental Epidemiologist, leader of interventional design, implementation, analysis, and reporting. All authors read and approved the final manuscript.

## Pre-publication history

The pre-publication history for this paper can be accessed here:

http://www.biomedcentral.com/1471-2458/13/945/prepub
